# Adaptation in Pathology

**Published:** 1897-07-31

**Authors:** 


### Medical Progress and Hospital Clinics.

[*The Editor will be glad to receive offers of co-operation and contributions from members of the profession. All letters should be addressed to* The Editor, at the Office, 28 & 29, Southampton Street, Strand, London, W.C.]

### ADAPTATION IN PATHOLOGY.

In an address delivered before the Congress of American Physicians and Surgeons, lately held in Washington, Dr. Welch considered a very interesting question in regard to pathological processes, namely, how far were they to be looked upon as instances of adaptation, analogous to those processes by which plants and animals of all kinds endeavour, successfully or not as the case may be, to fit themselves to changed surroundings.

In regard to this he brings forward many instances to show that what we call disease does, without doubt, lead to some form of adaptation to the new conditions involved—the so-called compensatory or work-hypertrophies being pointed to as good examples—but it is quite another thing to argue that the body is supplied with special protective mechanisms against different diseases. The question is whether this adaptation to new conditions which clearly manifests itself in more or less perfection in the progress of disease, works by the exercise of latent powers, special protective mechanisms, which animals possess but which they may, even for generations, never use; or, on the other hand, whether the power of adaptation does not come from the exercise of properties which the tissue-cells possess for ordinary physiological uses. The latter is the view taken by Dr. Welch.

If we look at a complete and fully-developed inflammation it is indeed hard to see anything conservative or protective in its results. "How," says Dr. Welch, "can one conceive of any useful purpose to the patient served by filling the air cells of his lung with pus cells, fibrin, and red corpuscles in pneumonia, or bathing the brain and spinal cord in serum and pus in meninigitis?" The fact would seem to be that the complete process which we call inflammation is not in itself an instance of adaptation, but is to a large extent an indication that adaptation has in that instance failed. The rush of blood, the accumulation of leucocytes, the effusion of serum are merely momentary when they are successful. Where they persist there it may be taken that the intruding irritant (organisms or what not) has got the upper hand so far as individual cells are concerned. The victory "is not always with the cells and other defensive weapons of the body. The struggle may be prolonged, may be most unequal, may cover a large territory, and the characters and extent of the inflammation furnish an index of these different phases of the battle." The inflammation, the heat, the redness, the effusion, the cell proliferation, are the index of the losses in the fight rather than of the fight itself, for when this is successful it takes place without showing much sign. Inflammation then is truly an adaptation to a morbid state. Not, however, an adaptation to the presence of intruding micro-organisms, but to the presence of dead and injured tissues, the tissues wounded in the contest.

The result, then, at which Dr. Welch arrives is that such examples of adaptation as we meet with in pathology present all degrees of fitness. Some are admirably complete; more are adequate, though but far from perfect; many are associated with such disorder and failure that it becomes difficult even to detect the element of adaptation. In any case, however, the agencies by which useful adaptations are brought about "are such as exist primarily for physiological uses, and while these may be all that are required to secure a good pathological adjustment, often they have no special fitness for this purpose."

The moral of all this is that there is much left for the physician. The agencies employed by nature may be all that can be desired; they may, however, be inadequate, even helpless, and their operation may but add to existing disorder. There is then ample scope for the exercise of skill in the treatment of disease.

